# Global Transcriptome and Weighted Gene Co-expression Network Analyses of Growth-Stage-Specific Drought Stress Responses in Maize

**DOI:** 10.3389/fgene.2021.645443

**Published:** 2021-01-26

**Authors:** Songtao Liu, Tinashe Zenda, Anyi Dong, Yatong Yang, Nan Wang, Huijun Duan

**Affiliations:** ^1^State Key Laboratory of North China Crop Improvement and Regulation, Hebei Agricultural University, Baoding, China; ^2^North China Key Laboratory for Crop Germplasm Resources of the Education Ministry, Hebei Agricultural University, Baoding, China; ^3^Department of Crop Genetics and Breeding, College of Agronomy, Hebei Agricultural University, Baoding, China

**Keywords:** hub gene, drought stress, RNA-seq, weighted gene co-expression network analyses, *Zea mays* L

## Abstract

Drought is the major abiotic stress threatening maize (*Zea mays* L.) production globally. Despite recent scientific headway in deciphering maize drought stress responses, the overall picture of key genes, pathways, and co-expression networks regulating maize drought tolerance is still fragmented. Therefore, deciphering the molecular basis of maize drought tolerance remains pertinent. Here, through a comprehensive comparative leaf transcriptome analysis of drought-tolerant hybrid ND476 plants subjected to water-sufficient and water-deficit treatment conditions at flared (V12), tasseling (VT), the prophase of grain filling (R2), and the anaphase of grain filling (R4) crop growth stages, we report growth-stage-specific molecular mechanisms regulating maize drought stress responses. Based on the transcriptome analysis, a total of 3,451 differentially expressed genes (DEGs) were identified from the four experimental comparisons, with 2,403, 650, 397, and 313 DEGs observed at the V12, VT, R1, and R4 stages, respectively. Subsequently, 3,451 DEGs were divided into 12 modules by weighted gene co-expression network analysis (WGCNA), comprising 277 hub genes. Interestingly, the co-expressed genes that clustered into similar modules exhibited diverse expression tendencies and got annotated to different GO terms at different stages. MapMan analysis revealed that DEGs related to stress signal transduction, detoxification, transcription factor regulation, hormone signaling, and secondary metabolites biosynthesis were universal across the four growth stages. However, DEGs associated with photosynthesis and amino acid metabolism; protein degradation; transport; and RNA transcriptional regulation were uniquely enriched at the V12, VT, R2, and R4 stages, respectively. Our results affirmed that maize drought stress adaptation is a growth-stage-specific response process, and aid in clarifying the fundamental growth-stage-specific mechanisms regulating drought stress responses in maize. Moreover, genes and metabolic pathways identified here can serve as valuable genetic resources or selection targets for further functional validation experiments.

## Introduction

Among all the abiotic stress factors that present threats to agricultural production, drought has the largest dramatic effect on crop growth and productivity, in both natural and manmade agricultural systems (Wheeler and von Braun, 2013). With the current evidence suggesting a continued increase in global warming, water shortage, and climate change, against a rising human population, crop breeders are faced with the biggest food security challenge in history (Hu and Xiong, 2014). It is estimated that the demand for agricultural products, including cereals, will increase by ∼50% by the year 2030, driven by population and income growth. This will require unprecedented sustained increases in the production of annual food crops (Farooq et al., 2009). Therefore, it is of top priority for crop breeders to develop drought-tolerant crop cultivars in order to sustain higher yields and global food security under the prevailing climate change scenario.

Globally, maize (*Zea mays* L.) ranks as the third staple crop after wheat (*Triticum aestivum* L.) and rice (*Oryza sativa* L.), contributing to both food security and industrial growth in some agro-based economies (Thirunavukkarasu et al., 2017). However, maize productivity and production expansion are negatively affected by drought stress, especially in the arid and semi-arid regions of South East Asia and Sub-Saharan Africa. More precisely, 60% of China’s maize production region lies in such drier regions. Consequently, a 20–30% maize yield loss per year occurs owing to water-deficit stress (Gong et al., 2014). Therefore, the development of maize hybrids with enhanced drought tolerance, either through conventional or genetic engineering approaches, is a priority goal for most maize improvement programs.

Maize is susceptible to drought stress throughout its life span, with the most devastating effects being experienced at the reproductive stage (Hu and Xiong, 2014). Generally, drought stress results in stomatal closure and reduced transpiration rates, decreased cell turgor, diminished photosynthetic efficiency, and overall plant growth (Zhang et al., 2018). The photosynthetic and gas exchange responses are the most sensitive to drought and the survival of drought-tolerant plants hinges on the maintenance of relatively high photosynthetic activity levels (Aslam et al., 2015). To cope with drought stress, plants institute several developmental-stage-specific changes at physiological and molecular levels. Numerous genes are expressed and translated in response to drought and have been identified to interact with the environment, thus the networks associated with water deficit conditions are quite complex. When plants are exposed to stresses, stress receptors, and transporters on cell membranes coordinate stress perception and signal transmission to the target genes. Then, phytohormones such as abscisic acid (ABA), cytokinin, auxin, and ethylene, etc., regulate numerous drought-inducible genes (Khan et al., 2019). At the same time, transcription factors (TFs), including basic region/leucine zipper motif (bZIP), NAM/ATAF/CUC transcription factor (NAC), myeloblastosis (MYB), WRKY, and dehydration responsive element binding protein (DREB) interact with *cis-*regulatory sequences to execute transcriptional regulation of gene expression, thereby providing adaptive responses to water-deficit conditions (Joshi et al., 2016). Additionally, plants activate cellular redox homeostasis maintenance through metabolic adjustment; transduce stress signals for the synthesis of defense enzymes and other antioxidant systems to protect cells from reactive oxygen species (ROS) damages; and institute stress-responsive proteins (Mahajan and Tuteja, 2005). Previous studies have highlighted the role of late embryogenic abundant (LEA) and heat shock proteins (HSPs) in enhancing tolerance to dehydration by functioning as chaperons to combat cellular damage (Hanin et al., 2011).

As a result of the fast advancement and reduction in the cost of next-generation sequencing (NGS) technologies, RNA-sequencing (RNA-Seq) has become a powerful tool for whole genome-wide gene expression profiling and has been widely used to investigate complex gene regulatory networks. This has immensely contributed to our better understanding of the complex molecular networks involved in adaptation and tolerance to water-deficit stress (Miao et al., 2017). RNA-seq technology has also been used in *Sorghum bicolor* L. (Fracasso et al., 2016) and rice (Ma et al., 2016). Resultantly, several genes that respond to drought stress have been identified (Zenda et al., 2019). In our previous study, we highlighted the role protein ubiquitination play in coordinating cellular crosstalk between stress and hormone signaling in maize seedlings under drought stress conditions (Zenda et al., 2019). Further, the co-expression of genes associated with osmotic adjustments and transporter proteins-maintained cell water balance at the seedling stage (Thirunavukkarasu et al., 2017).

Although global gene expression profiles in response to drought stress have been monitored in different maize tissues by micro-arrays and RNA-Seq experiments (Zheng et al., 2010; Min et al., 2016; Wang et al., 2018; Zenda et al., 2019), most of these studies were conducted separately in different tissues and at various developmental stages. Consequently, little is known about the gene co-expression networks of different developmental stages. In other words, it is not yet clear how maize drought adaptation is regulated genetically and how stress signaling pathways crosstalk with the developmental signaling pathways.

Fortunately, co-expression network analysis has become an important tool in the identification of gene co-expression in relation to their functional associations, this method identifies gene subsets that are highly correlated with each other within the network (Thirunavukkarasu et al., 2017). Particularly, the weighted gene co-expression network analysis (WGCNA), an approach in systems biology used to describe gene-related patterns in microarray samples allows for the amalgamation of vast amounts of microarray data from different biological samples and multiple experiments to obtain insights into genes from various metabolic pathways that possess similar expression patterns. The method has been successfully used to screen biomarkers and detect hub genes involved in metabolic pathways in abiotic stress responses (Wang B. et al., 2019).

Meanwhile, plant responses to abiotic stresses and drought stress, in particular, are dependent on the specific developmental stage and tissue affected and the level and duration of the stress. Therefore, the co-ordination of tissue-specific and developmental-stage-specific responses to the whole plant responses to drought stress needs to be considered (Wang B. et al., 2019). Thus, developing crops with higher resistance to water-deficit stress requires knowledge of the underlying physiological and molecular mechanisms of drought tolerance at various plant developmental stages.

Here, we utilized RNA-seq based approach to perform a comparative transcriptome analysis of growth-stage-specific drought stress responses between drought-tolerant maize hybrid Nongdan 476 (ND476) plants subjected to well-watered (control) and moisture-stressed (drought) conditions under short term conditions. Additionally, twenty-four RNA-Seq datasets were used to conduct WGCNA analysis in order to identify gene subsets possessing similar expression patterns and highly correlated with each other within different metabolic networks. Our results will be vital in clarifying the fundamental developmental-stage-specific molecular mechanisms regulating drought tolerance in the tolerant maize genotype.

## Materials and Methods

### Plant Materials and Drought Stress Treatment

The maize germplasm of hybrid ND476 that was used in this experiment was bred and provided by the North China Key Laboratory for Crop Germplasm Resources of the Education Ministry (Hebei Agricultural University, China). Maize hybrid ND476 is a highly drought-tolerant cultivar (as screened/identified by the Dryland Research Institute of Hebei Academy of Agricultural and Forestry Sciences, China). The experiment was conducted between May and July 2018 in a fully automated rain-proof shelter at Qing Yuan Experimental Station, Baoding, China (115.56 E; 38.80 N; 118 m). Each experimental plot measured 25 m^2^ (5 m × 5 m), with 60 cm × 30 cm plant spacings. The field arrangement was set up in a randomized complete block design, with the water-sufficient and water-deprived groups replicated three times. The soil water content was kept between 70 and 80% in the well-watered plots (control) and 15–20% in water-stressed plots (treatment). The soil water content across the water/drought treatments at all the four maize growth stages was consistently kept the same. This was made possible by the use of drip irrigation for agricultural water supply for the trials evaluated under both conditions (Wang N. et al., 2019). The relative soil water content of one meter underground was monitored by the TZS-1 soil moisture measurement instrument (Zhejiang Tuopu Technology Co., Ltd., Zhejiang, China). To prevent the transverse infiltration of soil moisture, building waterproof membranes of one-meter depth were put between control and treatment units.

**FIGURE 1:**
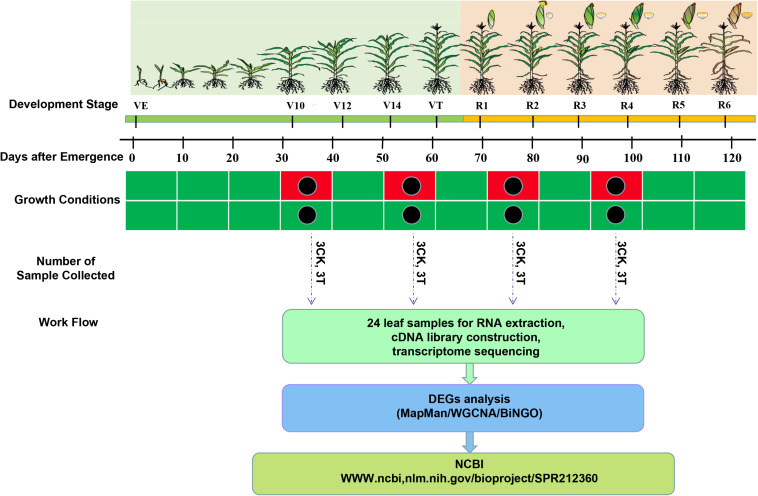
Schematic representation of the leaf transcriptome sequencing experiment under control and drought stress treatment in drought-tolerant hybrid line ND476 from vegetative to reproductive development stages. On the crop development stage timeline, green and yellow colors represent vegetative and reproductive stages, respectively. On growth condition timelines, green color represents water-sufficient (control), whereas red color represents water-deficit (drought) treatment periods, respectively. Black dots show the time of leaf sample collection after 12 days of treatment exposure.

Drought treatment was instituted at four different maize growth stages. Plants were water-deprived (a) from eight fully-expanded-leaf (FEL) to twelve FEL (V12) period (flared stage); (b) from twelve FEL until the tassel was visible (VT) (tasseling stage); (c) from self-pollination until 12 days post pollination (DPP), that is, the prophase of grain filling stage (R2); and (d) from 13 DPP until 24 DPP, that is, the anaphase of the grain filling stage (R4) (Figure 1). For each growth stage, leaf tissues were collected from the flag leaves of three replicates, of both control and drought treatment conditions. All the leaf samples were immediately frozen in liquid nitrogen and then stored at −80°C for further analysis.

### Determination of Photosynthetic Rate of Maize Leaves

Physiological parameters were measured for the maize under well-watered and water-deprived conditions from V12 to R2 stages. Specifically, photosynthetic rate (Pn) was measured according to drought treatment time and weather conditions using Li-6400 portable photosynthesis system (LI-COR Biosciences Inc., Lincoln, NE, United States) from 9:00 to 11:00 in the morning, as well as the conditions for measurement, were set as follows: photosynthetic photon flux density, 1500 μmol m^–2^ s^–1^; and chamber CO_2_ concentration, 300 μmol s^–1^. The student’s *t*-test was used to detect any significant differences in the data measured between control and drought treatment at each time point.

### Total RNA Extraction, cDNA Library Construction, and Transcriptome Sequencing

The total RNA was extracted from the leaf samples of the control and water-deprived plants using TRIzol reagent (Invitrogen, Carlsbad, CA, United States) following the manufacturer’s protocols. Subsequently, RNA was treated with DNase I (QIAGEN, Pudong, Shanghai, China) to eliminate contaminating genomic DNA. RNA degradation and contamination (integrity) were monitored on 1% agarose gels, then RNA quality was determined by 2100 Bioanalyzer (Agilent) and quantified using the ND-2000 (NanoDrop Technologies Inc., Wilmington, DE, United States). A total amount of 1 μg RNA per sample was used as input material. Only a high-quality RNA sample (OD260/280 = 1.8∼2.2, OD260/230 ≥ 2.0, RIN ≥ 6.5, 28S:18S ≥ 1.0) was used to construct the sequencing library. RNA purification, reverse transcription, library construction, and sequencing were performed at Shanghai Majorbio Bio-pharm Biotechnology Co., Ltd (Shanghai, China) according to the manufacturer’s instructions (Illumina, San Diego, CA, United States). RNA-seq transcriptome library was prepared following TruSeqTM RNA sample preparation Kit from Illumina (San Diego, CA, United States) using 1 μg of total RNA. Briefly, mRNA was isolated according to the polyA selection method by oligo (dT) beads and then fragmented by fragmentation buffer firstly. Secondly, double-stranded cDNA was synthesized using a SuperScript double-stranded cDNA synthesis kit (Invitrogen, Carlsbad, CA, United States) with random hexamer primers (Illumina). The short fragments (200 – 300 bp) were ligated with adapters and the suitable fragments were chose PCR amplified using Phusion DNA polymerase (NEB) for 15 PCR cycles. After quantified by TBS380 (Turner Biosystems, United States), the paired-end RNA-seq sequencing library was sequenced with the Illumina Novaseq 6000 (2 × 150 bp read length). Four cDNA libraries were prepared using mRNA isolated from the leaves of both water-deprived and well-watered maize plants of drought-tolerant hybrid ND476 at four developmental stages. The libraries were denoted ND1_Con (NDCa, NDCb, NDCc) and ND1_Tre (NDDa, NDDb, NDDc) (the leaves of control and treatment maize at V12 stage), ND2_Con (NDCA, NDCB, NDCC) and ND2_Tre (NDDA, NDDB, NDDC) (the leaves of control and treatment maize at VT stage), ND3_Con (NDC1, NDC2, NDC3) and ND3_Tre (NDD1, NDD2, NDD3) (the leaves of control and treatment maize at R2 stage), ND4_Con (NDC4, NDC5, NDC6) and ND4_Tre (NDD4, NDD5, NDD6) (the leaves of control and treatment maize at R4 stage). To identify genes responsive to drought stress in maize leaves at various growth stages, global gene expression profiling was performed by Illumina RNA sequencing of these libraries.

### Processing, Mapping of Sequencing Reads, and Gene Expression Quantification

Raw data (raw reads) generated by the Illumina Novaseq 6000 system were initially processed by SeqPrep^1^ and Sickle^2^ with default parameters. After trimming the adapter sequencing, removing low-quality bases, and filtering short reads, clean reads were separately aligned to the reference genome (ZmB73_Ref-Gen_v4) with orientation mode using TopHat (version 2.1.1)^3^ software. The mapping criterion was as follows: sequencing reads should be uniquely matched to the genome allowing up to 2 mismatches, without insertions or deletions. At the same time, Q20, Q30, GC-content, and sequence duplication level of the clean data (clean reads) were calculated. These high-quality reads were used in all the subsequent analyses. Subsequently, the gene expression level of each transcript was calculated according to the FPKM (fragments per-kilobase of the exon model per million mapped reads) based on the length of the reads count mapped to this transcript. For functional annotation, the quality reads were used for BLASTX alignment and annotation against non-redundant protein sequence database (Nr)^4^, Swiss-port (a manually annotated and reviewed protein sequence database)^5^, Clusters of Orthologous Groups (COG)^6^, the Kyoto Encyclopedia of Genes and Genomes (KEGG)^7^ and Gene Ontology (GO)^8^ with the threshold *e*-value = 1E-5.

### Differentially Expressed Genes (DEGs) Detection and Function Annotation of DEGs

Differential expression analysis of two samples was performed using the EdgeR package (Empirical Analysis of Digital Gene Expression in R)^9^. A differential expression analysis between stages was conducted using the ratio of FPKM values, and the *p*-value of each contrast corrected for multiplicity using the Benjamini and Hochberg method (Lei et al., 2015). In this study, genes with fold change (FC) ≥ 1.5 and *p*-value < 0.05 found by EdgeR were assigned as DEGs.

To further characterize DEGs in response to drought stress, the DEGs were visualized using the Mercator web tool subsequently loaded into MapMan software for a functional and categories annotation. The mapping file was used for visualizing the functional classes and pathways belonging to hierarchical BINs and sub-BINs based on the putative function. Subsequently, WGCNA analysis was performed to establish the maize DEGs co-expression network using the free online platform – Majorbio Cloud Platform^10^. In a scale-free weighted gene network, a node corresponded to a DEG, and an edge was determined by the similarity expression profiles of paired genes calculated by Pearson correlation. We selected a soft threshold (β) 12 to construct the co-expression networks according to the adjacency matrix (Supplementary Figure 1). The other parameters were as follows: minModuleSize = 30, minKMEtoStay = 0.3, mergeCutHeight = 0.25. Clusters (Modules) were visualized using Cytoscape software (version 3.4.0). To further explore the modules‘ functions, BiNGO plugins of Cytoscape were used for GO enrichment analysis based on the hypergeometric test and Bonferroni correction method (FDR < 0.05). Following WGCNA analysis, hub-genes were detected as the top 10% DEGs with the highest hub scores (Miao et al., 2017).

### Quantitative Real Time-PCR (qRT-PCR) Analysis

To validate the Illumina sequencing data results, quantitative real-time PCR (qRT-PCR) was conducted on 24 RNA samples that were used in the preparation of sequencing libraries using a C1000 (CFX96 Real-Time System) Thermal Cycler (Bio-Rad). Twelve genes that co-expressed at two treatment stages were selected for qRT-PCR to verify the RNA-seq results. Specific primers for each DEG were designed according to individual gene sequences using Primer Premier 5 Designer (Premier Biosoft International, Palo Alto, CA, United States). The cDNA for qRT-PCR analyses was synthesized from 1 μg total RNA with HiFiscript cDNA Synthesis Kit (CWBIO, Beijing, China). QRT-PCR experiments were performed on a Bio-Rad iQ5 Thermo Cycler (Bio-Rad, Hercules, CA, United States) using 2 × Fast Super EvaGreen^®^ qPCR Mastermix (US Everbright Inc., Daly City, CA, United States). Each PCR reaction mixture contained 10 μl of 2 × Fast Super EvaGreen^®^ qPCR Mastermix, 1 μl of template cDNA, 1 μl of forwarding primer (50 pmol), 1 μl of reverse primer (50 pmol), and 7 μl ddH_2_O to a final volume of 20 μl with three technical replicates of each gene. We used the maize gene *GAPDH* (accession no. X07156) as an internal control for data normalization. Additionally, a negative control was added. The relative mRNA abundance for each gene was determined for both the control and the drought-stressed samples by the 2^–ΔΔCT^ method (Livak and Schmittgen, 2001).

## Results

### Leaf Photosynthesis Rate Response of Maize Hybrid Cultivar ND476 to Drought Treatment

To determine whether the water-limited conditions could influence the physiological activities within the maize leaf tissues, in this research, we measured Pn of the drought-tolerant hybrid cultivar ND476 at different growth stages. Our analysis of the four stages showed that under well-watered conditions, at the V12 stage, Pn increased initially (from 1 to 9 days post-treatment exposure), and then decreased gradually thereafter. At the VT stage, Pn showed an increasing trend from day 1 onward. However, under well-watered conditions, at the R2 and R4 stages, Pn exhibited a slight gradual decrease throughout the treatment exposure period, starting from day 1 (Supplementary Figure 2). Meanwhile, all the four growth stages generally showed significantly reduced Pn under the water-deficit condition as compared to the well-watered condition (Supplementary Figure 2). This observation may indicate that with the increased drought exposure duration, leaf stomatal closure resulted in decreased leaf available CO_2_, or there was increased photo-oxidative damage induced by an accumulation of ROS.

### Illumina Paired-End Sequencing, Assembly and Annotation of Maize Leaf Transcriptomes

Resultantly, a total of 125.26 million raw reads were obtained. The raw sequencing data had been deposited in the NCBI Sequence Read Archive (SRA, Accession: SPR212360). After adaptors and low-quality reads were filtered out, 124.16 million clean reads were obtained, ranging from 23,029,648 to 72,636,578 for each sample. The clean reads were used for further analysis. Meanwhile, 20,432,633 (88.72%) to 64,399,057 (88.66%) clean reads were mapped onto unique positions on the maize reference genome (ZmB73_Ref-Gen_v4) (Supplementary Table 1). The Q30 base percentage and GC percentages exceeded 94.46% and 54.6%, respectively (Supplementary Table 1).

Subsequently, for functional annotation of the assembled transcriptome sequences, all the sequences were mapped onto the public genome database with an *E*-value threshold of 1e-5. We annotated 179,093 (93.1%) and 137,849 (71.66%) genes in the NCBI Nr database and the Swiss-Port protein database, respectively (Supplementary Table 2). Based on KEGG analysis, only 82,758 genes were successfully annotated, accounting for 43.02% of the total number. In addition, 169,691 (88.21%) and 133,525 (69.41%) genes were annotated using COG and GO databases, respectively (Supplementary Table 2).

Additionally, the similarities or differences of the twenty-four samples were analyzed using principal component analysis (PCA). The PCA results of all the samples showed a clear separation between treatment and control samples at different stages (Supplementary Figure 3). To measure the gene expression levels for the three replicates for each sample, Pearson correlation coefficients between samples were calculated. The results shown by way of heatmap revealed that each R2 between the two samples was higher than 90% except for one comparison (NDC4_vs_NDDb) (Supplementary Table 3). These results indicated the overall reproducibility and quality of the assay, which met the demands for further analysis.

### Identification of DEGs in Response to Drought

**FIGURE 2:**
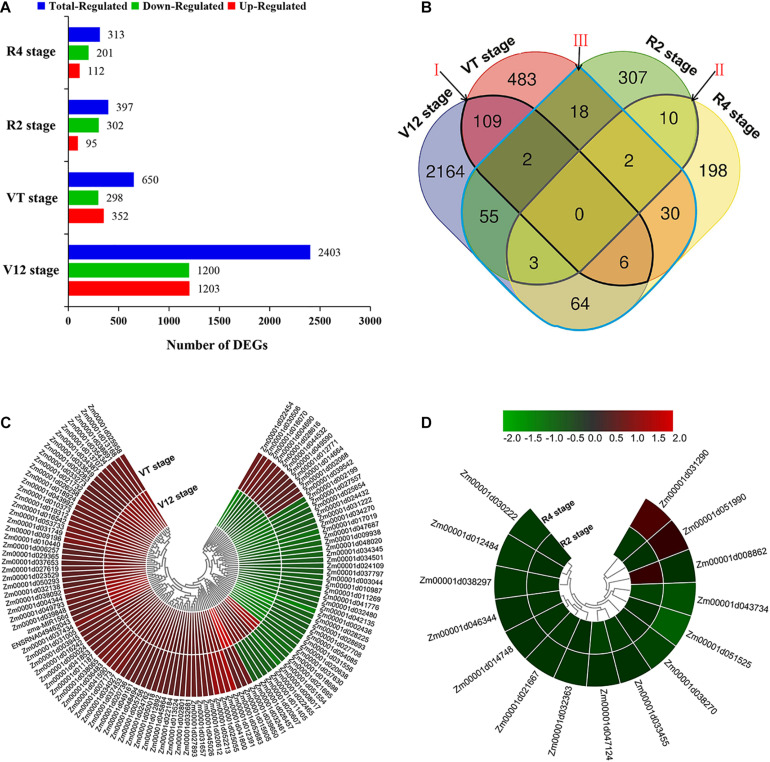
Gene differential expression and clustering analysis. **(A)** Number of DEGs expressed in different experimental stages **(B)** Venn diagram analysis of DEGs. The regions labeled Area I–III identify genes described under section ‘Identification of DEGs in Response to Drought’ above **(C)** Clustering analysis of DEGs common in vegetative stages (Area I). **(D)** Clustering analysis of DEGs common in reproductive development stages (Area II).

In order to reveal the transcriptional responses of maize leaves to water-stressed conditions, we compared the genes identified under water-sufficient and water-deficit conditions at four different growth stages. Gene expression levels were calculated and normalized to the RPKM values. Based on this analysis, a total of 3,451 DEGs were identified at four various maize growth stages. We obtained most numbers of DEGs at the V12 stage, including 1,203 upregulated and 1,200 downregulated. Meanwhile, 352 upregulated and 298 downregulated DEGs were identified at the VT stage. Similarly, we fished out 397 DEGs (95 upregulated and 302 downregulated) and 313 DEGs (112 upregulated and 201 downregulated) at the R2 and R4 stages, respectively (Figure 2A).

The number of DEGs showing overlaps and specific responses under drought stress in different growth stages is visualized in Figure 2B. A large number of DEGs were period-specific; there were 2,164, 483, 307, and 198 DEGs, respectively, at the four different stages. However, a limited number of common DEGs were detected. The area I represent 117 DEGs shared between V12 and VT stages after drought treatment, that is, the common DEGs identified in the two vegetative stages. Area II represents 15 DEGs shared between R2 and R4 stages after drought treatment, that is, the common DEGs identified in the two reproductive stages. There were 180 drought-responsive DEGs identified in the vegetative stages (V12 or VT) and also differentially expressed at the reproductive stages (R2 or R4) after drought treatment; that is, the DEGs identified at both vegetative and reproductive stages (Area III, Figure 2B).

To further understand the gene expressions between different stages, we performed the hierarchical clustering analysis of the identified DEGs (Figures 2C,D and Supplementary Figure 4). A total of 28 DEGs showed downregulated and 69 DEGs showed upregulated both of V12 and VT stages, but 20 DEGs showed the opposite trend of expression of the two stages (Figure 2C). Thirteen DEGs were downregulated both of R2 and R4 stages, 2 DEGs showed the opposite trend of expression of the two stages (Figure 2D). The others shared DEGs in different stages also showed different expression patterns (Supplementary Figures 4A–D). These results indicated that there were different mechanisms of maize drought stress responses at various growth phases.

### Functional Annotation of DEGs Using MapMan

**FIGURE 3:**
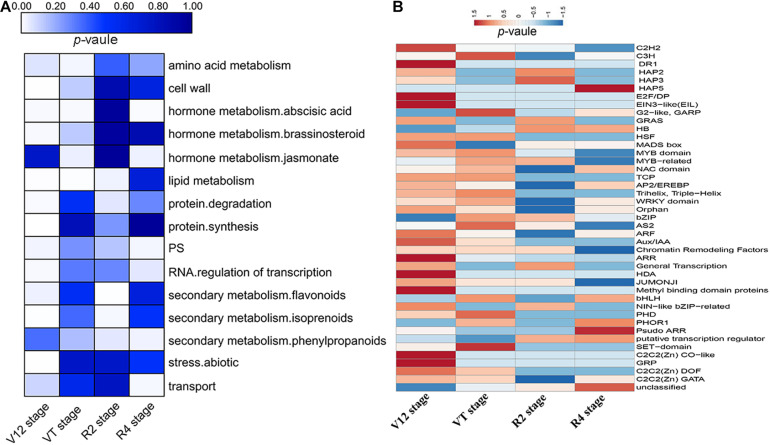
Enrichment analysis of the functional categories of DEGs for the four maize growth stages by MapMan. **(A)** Enrichment of DEGs into functional categories for different stages. **(B)** Enrichment of transcription factor subcategories.

All DEGs of four growth stages were assigned to MapMan functional categories. The DEGs were grouped into 35 BINs with putative functions (Supplementary Figure 5). We found out that 720, 183, 147, and 72 DEGs of the V12, VT, R2, and R4 stages, respectively, were not assigned to any functional group (BIN 35) due to lack of annotation information (Supplementary Figure 5). The DEGs of the V12 stage were mainly annotated to the cell wall, lipid metabolism, photosynthesis (PS), protein synthesis, and degradation, abiotic stress, secondary metabolites biosynthesis and hormone metabolism (Figure 3A). The highly enriched categories of the VT stage DEGs included lipid metabolism, amino acid metabolism, and hormone metabolism (Figure 3A). Meanwhile, the enriched categories of the R2 stage DEGs included lipid metabolism, protein degradation, and secondary metabolites biosynthesis, whereas the R4 stage DEGs related to transport, PS, hormone metabolism, secondary metabolites biosynthesis, and RNA transcriptional regulation were highly enriched (Figure 3A).

An overview analysis of DEGs was generated with the MapMan tool and the drought-inducible regulated genes were classified into different regulatory processes (Table 1). The DEGs involved in PS showed upregulated expression under drought stress conditions in all the stages, except the VT stage. A total of 171 DEGs related to transport were altered in expression among the four growth stages. Moreover, 266 protein kinases including serine/threonine-protein kinases, leucine-rich repeat receptor-like proteins, receptor-like kinases, phospholipases, and protein phosphatases were observed to be mostly upregulated at the vegetative stages, whilst showing downregulation at the reproductive stages. Several plant hormones, which functions as regulatory compounds, were identified to be responsive to drought stress including abscisic acid (ABA), auxin, brassinosteroids (BR), gibberellic acid (GA), salicylic acid (SA), and ethylene. DEGs related to auxin and SA were downregulated, whilst the DEGs involved in other hormones showed an increased expression under drought stress. Additionally, 289 differentially expressed TFs were identified, such as C_2_H_2_, bHLH, HB, MYB domain, MYB-related, and WRKY domain (Figure 3B). In the current study, more increased abundance TFs were identified in the vegetative stages compared to the reproductive stages.

**TABLE 1:** Classification of drought-response regulated DEGs into different categories according to MapMan annotation.

**Category**	**V12 stage**		**VT stage**		**R2 stage**		**R4 stage**	
	**Up-**	**Down-**	**Up-**	**Down-**	**Up-**	**Down-**	**Up-**	**Down-**
**Photosynthesis**								
Photosystem II	3	1	0	1	1	0	3	0
Photosystem I	2	0	0	3	0	0	1	0
Electron carrier (ox/red)	3	0	1	2	1	0	0	0
Calvin cycle	0	0	0	2	1	1	0	0
**Transport**								
Transport sugars	8	3	1	2	0	1	0	3
Transport amino acids	11	7	1	4	2	0	1	4
Transport ammonium	0	2	0	1	1	0	0	1
Transport phosphate, nitrate, sulfate	2	4	1	2	0	0	2	4
Transport metal	3	2	3	0	0	3	0	0
Transport peptides and oligopeptides	11	6	1	1	0	1	0	5
Transport potassium	8	4	2	0	0	3	1	1
ABC transporters	6	4	2	1	0	2	1	3
Transport misc	9	6	2	5	0	4	1	2
**Protein kinases and phosphatases**								
Serine/threonine-protein kinase	11	4	2	1	0	5	0	1
LRR receptor-like protein	27	6	10	1	1	7	1	2
Receptor-like kinase	28	20	5	7	0	5	0	10
Phospholipase	8	2	5	0	0	1	2	0
Calmodulin	10	6	0	2	0	4	0	1
Mitogen-activated protein kinase kinase kinase	2	1	1	2	0	0	0	0
Protein phosphatase	2	6	3	0	0	0	4	0
Protein phosphatase 2C	4	0	2	0	0	0	2	2
**Plant Hormones**								
Abscisic acid	14	2	8	1	1	3	9	3
Auxin	4	7	3	3	1	2	1	2
Brassinosteroid	11	4	4	0	0	0	1	1
Jasmonic acid	3	3	4	2	1	1	0	3
Salicylic acid	1	4	1	2	0	0	0	2
Gibberellic acid	3	0	0	0	1	1	0	1
Ethylene	6	4	2	2	2	2	0	0
**Transcription factors family**								
Basic Helix-Loop-Helix	9	3	4	2	2	1	1	1
C2H2 zinc finger	6	3	0	1	0	2	0	1
Homeobox transcription factor	11	4	2	0	0	1	2	2
MYB domain transcription factor	4	3	3	3	0	2	0	2
MYB-related transcription factor	3	1	0	1	0	1	0	1
NAC domain transcription factor	2	4	3	2	0	0	0	1
WRKY domain transcription factor	3	3	1	1	0	0	0	1
bZIP transcription factor	3	0	1	2	0	3	1	1
G2-like transcription factor	2	0	1	3	0	1	0	2
Other transcription factor families	42	54	22	16	7	11	11	3
**DEGs related to detoxification**								
Thioredoxin	2	1	0	1	0	0	2	0
Glutathione S-transferases	6	3	1	4	0	1	0	2
Peroxidase	8	6	1	3	1	4	0	2
Ascorbate and glutathione	5	5	1	0	1	0	1	1
Glutaredoxins	5	1	0	1	0	0	1	0
**DEGs involved in defense**								
Heat shock proteins	4	20	8	7	0	1	0	0
Late embryogenesis proteins	6	0	2	1	0	0	0	0
Pathogenesis-related proteins	5	1	4	0	0	2	0	0
**DEGs response to abiotic**								
Response to heat	4	23	8	7	0	1	0	0
Response to drought/salt	6	4	3	0	0	0	2	0
Response to cold	3	1	0	2	0	2	0	2
**Secondary metabolism**								
Isoprenoids metabolism	13	2	5	0	3	0	0	5
Phenylpropanoids metabolism and biosynthesis	14	7	6	3	0	2	0	12
Flavonoids metabolism	9	15	1	2	7	1	2	4
Sulfur-containing metabolism	5	6	2	2	0	0	0	4

The DEGs were involved in maintaining redox homeostasis by a series of enzymatic compounds including thioredoxin (TRX), glutathione S-transferases (GST), and peroxidase (POD), which played major roles in protecting maize from oxidative damage. Additionally, 61 DEGs were annotated to stress defense. HSPs mainly showed upregulated, whist LEA and pathogenesis-related proteins (PRPs) had increased abundance under drought. The identification of such a great number of regulatory DEGs showed that there were multiple signaling mediators and intricate pathways in response to drought stress. Subsequently, we also obtained 10, 3, and 2 DEGs of the V12, VT, and R4 stages that were annotated to “response to drought/salt” (BinCode: 20.2.3) (Table 1). Among them, responsive to dehydration 22 (RD22, *AT5g25610*) mediated by ABA was identified in the V12 and VT stages and was involved in response to desiccation. Drought-responsive family protein *AT3g05700* and ERD (early-responsive to dehydration stress, *AT4g22120*) family protein may play a role in maize response to water-stress in the V12 stage. In addition, gene encoding AOC (allene oxide cyclase, *AT3g25780*), which is involved in jasmonic acid biosynthesis, is suggested to play a functional role in maize response to drought at the V12 stage. Taken together, these differentially expressed genes were speculated to be the vital cogs in maize drought stress tolerance, and hence aroused our keen interest for further discussion.

### Co-expression Network Analysis of DEGs by WGCNA

**FIGURE 4:**
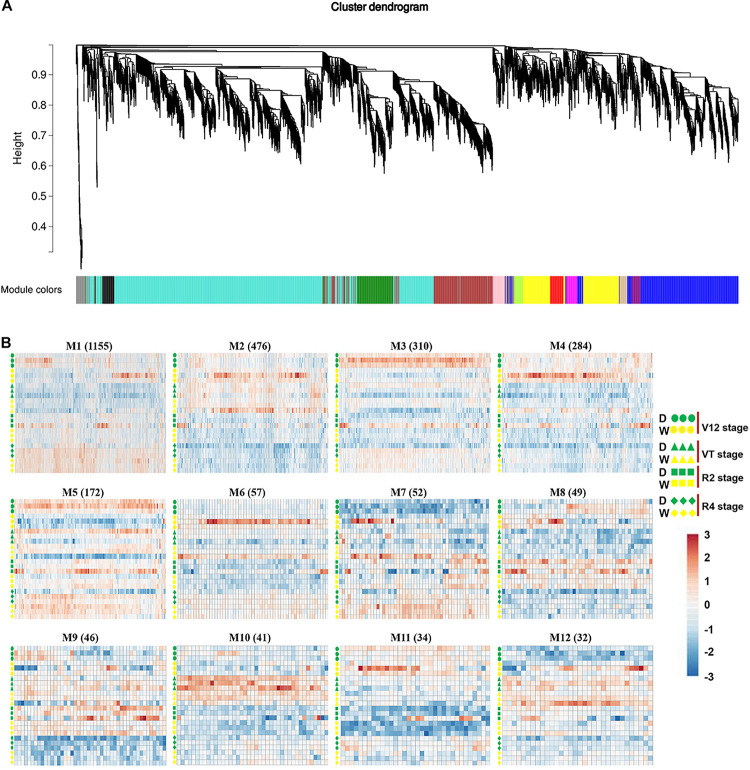
Co-expression network analysis identifying gene modules underlying maize drought stress response at four different growth stages. **(A)** Hierarchical cluster trees showing co-expression modules identified using WGCNA of the differentially expressed genes. **(B)** Heatmap showing gene expression levels of the genes within the 12 modules across four growth stages. W, well-watered; D, drought treatment.

**FIGURE 5:**
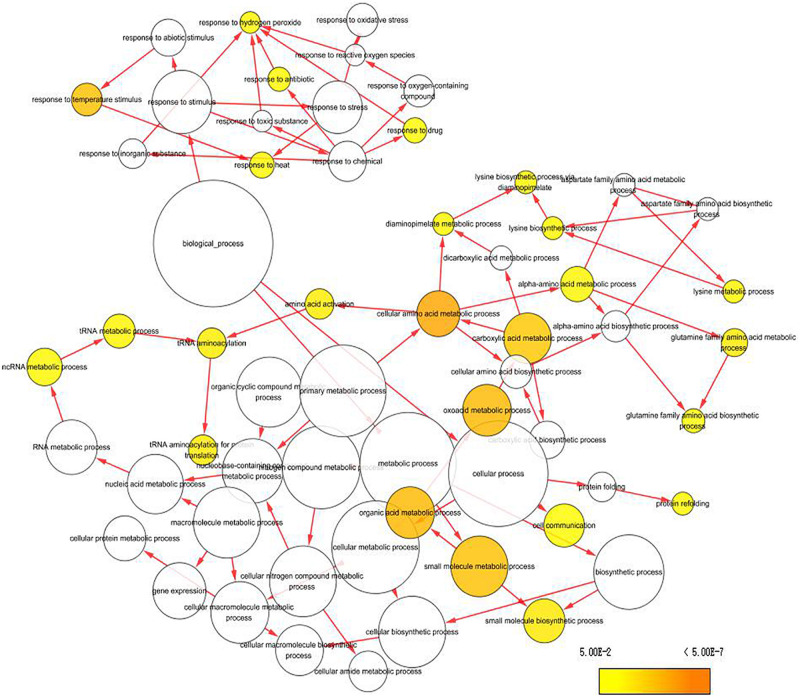
Enrichment analysis of DEGs clustered in Module 1 (turquoise module) using GO terms from GO Slim. Significantly over-represented GO terms were visualized by BiNGO application in Cytoscape. The size of a node represents the proportion of the GO term to the number of targets in GO biological process category. The deeper the color, the higher the level of significance.

To capture crucial shifts in gene networks in maize under water-stressed conditions, we further applied the WGCNA approach to perform a network-level analysis of co-expression relationships among 3,451 DEGs based on their expression patterns throughout the four growth stages. After filtering, a total of 2,771 DEGs were divided into 12 modules (clusters) (designated M1-M12) comprising of 32 to 1,155 highly co-expressed genes (Figure 4, Supplementary Figure 6, and Supplementary Table 4). GO enrichment analysis of each module by BiNGO highlighted vital biological processes represented by a series of co-expressed genes. Module M1 formed the largest cluster of 1,155 DEGs enriched in functions related to metabolic processes (cellular amino acid, oxoacid, organic acid, and small molecule) and response to temperature stimulus (Figure 5 and Supplementary Table 5). We also observed that cluster M5 had 172 DEGs enriched in functions related to metabolism process (peptide, cellular amide, and protein) and biosynthetic process (peptide, amide, and cellular macromolecule) (Supplementary Table 5). Meanwhile, module M11, comprising a cluster of 34 DEGs, had its DEGs annotated to biosynthetic process (cinnamic acid, phenylpropanoid, and carboxylic acid) and metabolism process (cinnamic acid, aromatic amino acid, and benzene-containing compound) (Supplementary Table 5). Modules M2, M10 and M12 showed enrichment of GO terms related to photosynthesis (Supplementary Table 5). Additionally, module M10 represents DEGs that showed high-expression specifically in the VT-stage and were enriched in GO terms associated with ion homeostasis and transport (Supplementary Table 5). The black module M7 included 52 DEGs involved in response to an abiotic or environmental stimulus (Supplementary Table 5). Further, the brown module M3 included DEGs related to ribosome and ribonucleoprotein complex biogenesis, gene expression, and RNA processing (Supplementary Table 5). Modules M4, M6, M8, and M9 did not get enriched in any GO term. By combining our gene expression pattern and GO enrichment analysis results, we concluded that the DEGs mainly participated in metabolic and biosynthetic processes, photosynthesis, ion homeostasis, transport, and response to abiotic stimulus under drought stress conditions.

### Identification of Hub Genes Within Network Modules

**FIGURE 6:**
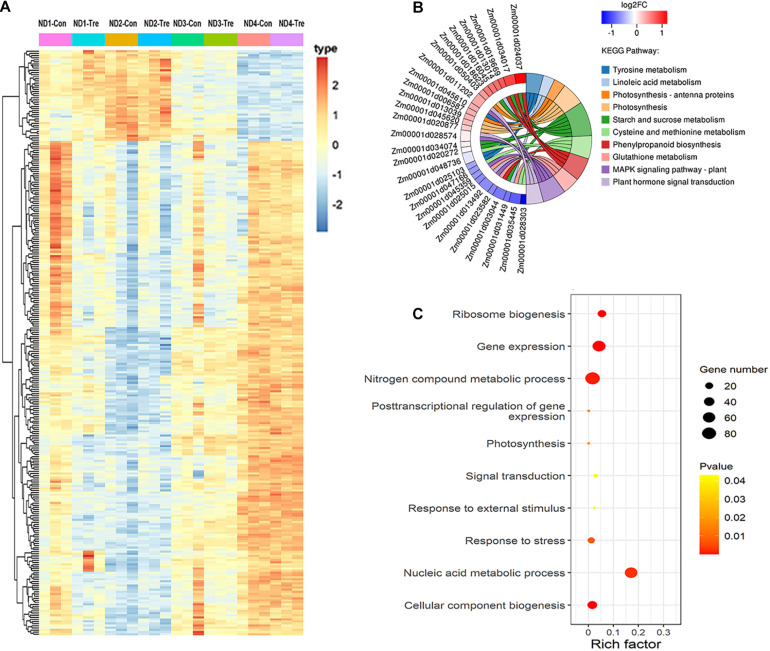
Co-expression network analysis of hub-genes. **(A)** Heatmap showing the hub-genes that were expressed at four different maize growth stages. **(B)** KEGG pathway enrichment analysis of the hub-genes. **(C)** GO functional classification of the hub-genes.

There were some genes with extremely high connectivity with other genes, and these were designated as hub genes in each network module. Owing to their central location within the network clusters, the hub genes were considered to be vital components of the networks. Selecting only the top 10% of genes that showed high connectivity degree, a total of 277 DEGs were identified as hub genes (Figure 6A), including 17 TFs represented from distinct families including WRKY, MYB-related, C_2_H_2_, MYB, and NAC TFs (Supplementary Table 6). Seven hub genes were also identified as crucial enzymes playing a key role during maize drought stress response (Supplementary Table 6). Besides TFs and enzymes, two HSP90 genes that were also observed to respond to water-deficit stress conditions. Six hub-genes (*Zm00001d005410*, *Zm00001d025920*, *Zm00001d008462*, *Zm00001d019363*, *Zm00001d020272*, and *Zm00001d047235*) were observed to respond to drought stress by taking part in photosynthesis (Supplementary Table 6). Further, we conducted significant KEGG pathway enrichment analysis of these hub genes by using the hypergeometric test. Resultantly, by comparing the top ten pathways that were most enriched in the hub-genes, we discovered that starch and sucrose metabolism (6 genes), photosynthesis (4), linoleic acid metabolism (2), and photosynthesis - antenna proteins (2) were dominant under droughts stress conditions (Figure 6B and Supplementary Table 7). Moreover, the significantly enriched GO terms related to drought response were identified, including photosynthesis (GO: 0015979), response to stress (GO:0006950), and response to external stimulus (GO:0009605) (Figure 6C).

### Validation of DEGs by Quantitative Real-Time PCR (qRT-PCR)

**FIGURE 7:**
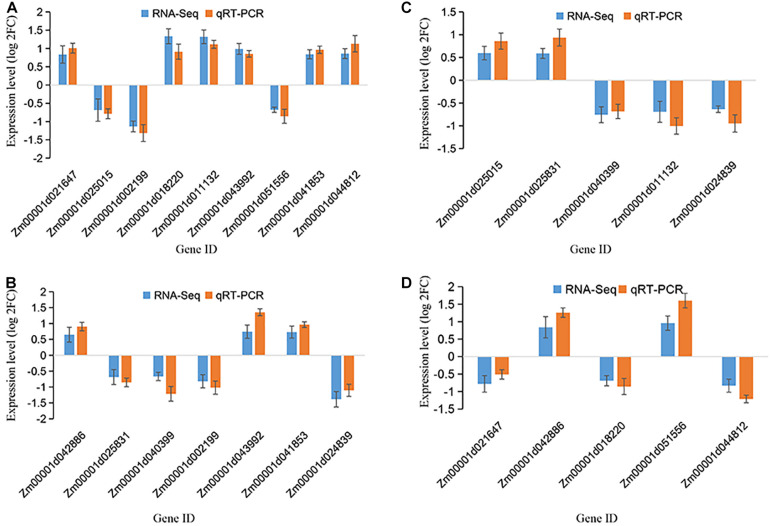
qRT-PCR validation of selected genes among various maize growth stages. The *y*-axis represents the gene relative expression levels (fold changes) in real-time PCR analysis and RNA-seq data. **(A)** The DEGs identified at V12 stage; **(B)** The DEGs identified at VT stage; **(C)** The DEGs identified at R2 stage; **(D)** the DEGs identified at R4 stage. All genes with negative values means they were downregulated. Maize gene GAPDH (accession no. X07156) was used as the internal reference.

To confirm the accuracy of the RNA sequencing results, we conducted a validation experiment by qRT-PCR analysis for three biological replicates. The representative DEGs were chosen based on them being highly differentiated in response to drought and reported to be related to drought resistance. Precisely, the patterns of RNA-Seq expressions on all the 12 DEGs were consistent with the qRT-PCR data, suggesting that the patterns of the RNA-seq expression on all the sampled genes were replicated by the qRT-PCR approach (Figure 7 and Supplementary Table 8). A correlation coefficient (R^2^) (of the fold changes before and after drought treatment) of 93.01% was obtained (Supplementary Figure 7), endorsing our RNA-Seq data as reliable.

## Discussion

Drought stress during the transition from vegetative to reproductive development greatly affects grain production in maize (Zheng et al., 2010; Aslam et al., 2015). Thus, a full understanding of physiological, biochemical, and gene regulatory networks associated with water-deficit stress tolerance at these different growth stages in maize becomes imperative for breeding drought-tolerant cultivars. However, the complex adaptive mechanisms underpinning water-deficit stress tolerance from vegetative growth to reproductive development have remained elusive despite recent advances in molecular biology approaches (Bhanu et al., 2016). Therefore, in this report, we have employed RNA-seq based approach to perform a comprehensive comparative transcriptome analysis of drought-tolerant hybrid ND476 from the vegetative to reproductive growth stages to identify key regulatory genes and gene co-expression networks involved in maize drought stress response. We have further conducted photosynthetic parameter measurements to support the RNA-seq data. Additionally, functional validation by qRT-PCR analysis corroborated the differential expression of these identified genes. Our findings not only enrich our knowledge about maize drought stress tolerance mechanisms but also provide a valuable genetic resource or selection target for the genetic improvement of maize.

### Photosynthesis Related Genes Were Differentially Altered in Response to Drought

Compared to other cereal food crops, maize is relatively sensitive to water-deficit stress (Pinheiro and Chaves, 2011). Photosynthesis is the most sensitive physiological process of plants subjected to abiotic stresses (Zhou et al., 2019). A stress-induced negative effect on any of the components of the photosynthesis systems may lead to a reduction in the overall photosynthetic performance (Lamaoui et al., 2018). To adapt to water-deprived conditions, plants will immediately close the stomata, thereby reducing the leaf gas exchange. This has negative influences on photosynthetic parameters. In the current investigation, our physiological analysis results showed that photosynthesis rate was significantly repressed by drought stress (Supplementary Figure 2). At the molecular level, MapMan annotation and analysis results of the common elements of modules M2, M10, and M12 by BiNGO found out that photosynthesis was significantly enriched mostly at the V12 and R4 stages in response to drought stress. Furthermore, we found several genes in the photosynthesis pathway with altered transcription abundance (Table 1 and Supplementary Figure 5). The photosynthesis-related genes showed downregulated expression at the VT stage but showed upregulation at the V12 and R4 stages (Table 1). This is consistent with the measured photosynthesis rate, and showing that drought-tolerant hybrid ND476 exhibited the well-maintained level of photosynthesis under drought stress conditions. Previously, Wang B. et al. (2019) observed that drought stress at the V9-V10 stages decreased net Pn in maize resulting in abnormal ear primordium development. Several other reports (Min et al., 2016; Lamaoui et al., 2018) have shown that drought stress represses photosynthesis in maize plants. However, Ma et al. (2016) found photosynthesis-related genes displaying upregulated expression in rice drought-tolerant line IAC1246, but the downregulated expression in rice drought-sensitive line IRAT109. Taken together, our results suggest that drought stress retards photosynthetic efficiency in plants, hence strategies aimed at improving photosynthesis under drought conditions can be vital for plants’ survival.

### Stress Signal Transduction and Protein Kinases Under Drought Stress Conditions

Drought-tolerance is typically a complicated trait since drought stress affects multiple aspects of plant physiology and metabolism, consequently, changing thousands of genes’ expression (Min et al., 2016). At the initial stage of stress signal transduction, receptors, and transporters on cell membranes perceive stress bridging the gap between perception and transmission of the signals to the target genes and contributing to plant survival. Water-limited conditions are often related to a significant increase in transport-proteins and channel proteins (Guo et al., 2006). In this study, several transporters were identified in maize response to drought stress conditions, including phosphate transporters, ion transporters and nitrate transporters (Table 1). Additionally, receptor kinases, another vital type of membrane protein, were found differentially expressed in response to drought stress, including serine/threonine-protein kinases, cysteine-rich receptor-like protein kinases, and proline-rich receptor-like protein kinases (Table 1). Among them, cysteine-rich receptor-like protein kinase (*Zm00001d008462*), proline-rich receptor-like protein kinase (*Zm00001d043480*) and receptor-like serine/threonine-protein kinase (*Zm00001d002199*) was identified as hub-genes (Supplementary Table 6). Receptor-like protein kinases (PLPKs) form the largest part of all plant kinases, and are well-known for playing an important role in abiotic stress responses. Several PLPKs detected in both leaves and roots of Bothriochloa ischaemum significantly changed their expression in response to drought stress (Li et al., 2019).

Protein kinases (PKs) are sensor responder genes which initiate phosphorylation cascades and play essential parts in water-deficit responses (Singh and Laxmi, 2015). In this current study, PKs were differentially expressed to regulate stress signaling transmitting in maize under water-limited conditions (Table 1). Moreover, serine/threonine protein phosphatase 2A (Zm00001d019363) was identified as a hub-gene (Supplementary Table 6). Similarly, the identical PKs were reported in faba bean (Vicia faba L.) drought-tolerant variety hassawi-2 response to drought stress (Khan et al., 2019). From this discussion, we can infer that a complex web of signaling was triggered under water-stressed conditions, which relayed messages through the plasma membrane to the cell, activating a signal transduction cascade. Then, TFs were modulated by the PKs, as a result affecting corresponding response to the downstream drought-responsive genes that enabled maize to regulate its growth and metabolism under drought stress (Wang et al., 2016).

### Enhanced Cellular Redox Homeostasis May Contribute to Drought Tolerance in Maize

When plants are subjected to water-stressed conditions, there is a rapid and transient production of ROS which can damage cellular components and structures. In response, plants institute various mechanisms to re-establish the cellular redox balance and homeostasis, and avoid cellular components and structures damage caused by ROS. Cellular redox homeostasis transduced signals for the synthesis of defense and antioxidant enzymes contribute to the modification of the antioxidant system and cell turgor maintenance by osmotic adjustment (Mahajan and Tuteja, 2005). In the current study, twenty-five peroxidases, six TRXs, fourteen L-ascorbate peroxidase (APX), and seventeen GSTs were observed to be differentially altered in their expressions in response to drought stress at various maize growth stages (Table 1).

It is well known that peroxidases are central in neutralizing the damaging effects of toxic peroxides and other ROS that accumulate under oxidative stresses. It has been reported that the upregulated expression of peroxidases protected wheat plants from ROS-induced cell damage under drought stress (Sheoran et al., 2015). Similarly, Khan and Komatsu (2016) indicated peroxidases’ essential role in soybean root ROS scavenging and cellular redox homeostasis. The chloroplast thioredoxin (TRX) systems compose an important component of the redox network, with thioredoxin reductase (TRs) functioning in re-establishing cellular redox homeostasis. Previous researchers have identified that TRX genes were upregulated or downregulated in response to drought stress, adjusting the cellular redox status in the process (Xie et al., 2016). Ascorbate peroxidases (APXs) are a vital cog of the complex stress response network. APXs play a role in detoxifying hydrogen peroxide (H_2_O_2_) in the chloroplasts and cytosol using ascorbate as a substrate (Xu and Huang, 2010). APXs expression has been reported to be significantly increased in winter rapeseed (*Brassica napus* L.) under drought stress conditions (Urban et al., 2017). Additionally, a previous study identified APXs being involved in ROS scavenging in maize in response to water-deficit stress (Miao et al., 2017). Glutathione-S-transferases (GSTs) are conjugating enzymes involved in the detoxification of a wide range of harmful substances such as ROS or xenobiotic compounds (Xu and Huang, 2010). Accumulation of GST in wheat has also been reported under drought stress conditions (Bazargani et al., 2011). However, GST showed downregulation in rapeseed subjected to drought stress (Mohammadi et al., 2007). Overall, our results here indicate that hybrid cultivar ND476 could endure water stress via increased activation of genes associated with ROS detoxification and oxidation-reduction processes, whereas the downregulated expression of some stress redox homeostasis genes may imply the complexity of the cell redox system in drought stress response.

### Regulation of Drought Stress by Transcriptional Factors

As gene regulators, TFs play a key role in modulating gene expression and transmitting stress signals in plant cells. Therefore, TFs have been designated master regulators of abiotic stresses, including drought (Wang et al., 2016). In the current study, there were 42 classes of TFs that were annotated by MapMan among the four maize growth stages (Figure 3B). Meanwhile, among the total 277 hub-genes, 17 (6%) were annotated as TFs, belonging to 13 different families (Supplementary Table 6), indicating that differential transcription mechanisms function in the water stress signal transduction pathway in maize. A large number of identified TFs belong to MYB, NAC, WRKY, and bZIP families, which are well known for their roles in drought stress response (Thirunavukkarasu et al., 2017). Previously, Tran et al. (2004) observed that three *Arabidopsis* genes (ANAC019, ANAC055, and ANAC072) and two homologous maize NAC transcripts were abiotic-stress-inducible-expression genes. NACs were identified in foxtail millet (*Setaria italica* L.) (Shi et al., 2018) and maize (Song et al., 2017) responding to drought stress. WRKY TFs were identified as the key drought response elements by changing their differential expressions under drought stress (Yan et al., 2014). MYB factors were related to hormone signal transduction and abiotic stress response (Liu et al., 2015). Overexpression of R1R2R3-MYB TF in *Arabidopsis* significantly enhanced the tolerance of transgenic plants to drought stress (Zhang et al., 2019). More recently, Wu et al. (2019) reported that over-expressing ZmMYB3R enhanced drought and salt stress tolerance in transgenic maize. A previous study by Song et al. (2017), observed that C_2_H_2_ and bHLH TF factors were involved in drought stress response in maize leaves. Additionally, Zhang et al. (2014) observed that several NACs, MYBs, bZIPs, bHLHs, and other TFs expression was tightly coupled to plant water potential, indicating their involvement in *Medicago truncatula* L. drought adaptation responses. Furthermore, 49 TFs from bHLH, bZIP, C_2_H_2_, MYB, and NAC families were found in maize spatio-temporal drought stress response (Miao et al., 2017). Taken together, the complex expression changes of these TFs crucially contribute to the drought stress tolerance of maize hybrid line ND476 as these TF genes interact with other molecular actors in complex networks.

### Genes Related to Hormone Signaling Are Critical for Drought Stress Response

Plant hormones participate in numerous plant abiotic stress responses. For instance, a large number of genes related to biosynthesis or signaling of plant hormones such as auxin, ABA and ethylene were identified in a drought-tolerant faba bean variety under drought stress (Khan et al., 2019). Here, several auxin-responsive genes were altered in their expression levels in response to drought stress (Table 1). Previously, a series of auxin-responsive genes were induced by several abiotic stresses, revealing that auxin may function in abiotic stress signaling. Consistent with our findings, genes encoding auxin-responsive protein exhibited significantly increased abundance under partial desiccation treatment (Jing et al., 2017). It is well recorded that ABA is a critical messenger that mediates the adaptive response of plants to abiotic stresses. When plants are subjected to drought stress, a large number of ABA-regulated genes are accumulated. Previously, water deprivation resulted in high levels of ABA in maize, which stimulated the production of ROS and regulated the activity of the antioxidant defense system (Jiang and Zhang, 2002). Similarly, in our current study thirty-two (78%) genes related to ABA biosynthesis were upregulated. However, nine *cis-*epoxycarotenoid dioxygenase (NCED3) enzymes showed decreased expression under drought treatment (Table 1). By comparing our results with the previous observations, we realized that NCED3, a vital enzyme in ABA biosynthesis, was also downregulated in faba bean battling drought stress (Khan et al., 2019).

Meanwhile, we observed that sixteen (76%) genes encoding brassinosteroid (BRs) biosynthesis had an increased abundance in response to drought (Table 1). The BRs have critical functions in detoxifying oxidative damage by the expression of genes involved in ROS scavenging under drought stress conditions. Previously, BRs have also been identified in maize response to water-deficit conditions (Fracasso et al., 2016). The stress-tolerance ability of BR rests with its crosstalk with other plant hormones such as ABA (Divi et al., 2010). Additionally, we identified ethylene-, jasmonic acid- and salicylic acid-regulated genes that were responsive to drought treatment, suggesting that these plant hormones may play critical roles in drought stress signaling.

### Stress Defense Genes Are Essential for Plant Response to Water-Stress Conditions

Expectedly, several genes involved in stress defense including HSPs, pathogenesis-related (PR) and LEA proteins showed significant changes in expression under drought stress conditions (Table 1). Two HSP90 genes (*Zm00001d041719* and *Zm00001d052809*) were identified as hub-genes in drought stress response. Meanwhile, twenty-eight HSPs exhibited decreased abundance at the four growth stages after drought treatment (Table 1). HSPs play important roles in helping proper folding or unfolding of proteins and preventing unwanted aggregation, as well as contributing to cellular homeostasis in cells under stress conditions (Wang et al., 2004). Here, the downregulation of the HSPs at the V12 stage may imply that short-term drought stress had a significant negative impact on the chaperon activities of these protective proteins at this stage. PRs are also thought to be involved in plants’ developmental processes and defense responses against abiotic stress (Kim et al., 2008). In our research, unlike HSPs, PR proteins showed significantly increased accumulation at the V12 and VT stages (Table 1). Previously, drought stress-induced response of PR proteins showed increased abundance in tobacco (Gharechahi et al., 2015) and grapevine (Król and Weidner, 2017).

Late embryogenic abundant proteins are commonly induced during water-deficit conditions, and their expressions are regulated by ABA and C_2_H_2_ TFs, helping to maintain the osmotic balance (Thirunavukkarasu et al., 2017; Khan et al., 2019). LEA proteins were induced in vegetative tissues by drought stress. Several LEA proteins showed increased abundance both at the V12 and VT stages in response to drought stress (Table 1). The relation of this protein with drought stress has been previously reported in wheat (Li et al., 2012) and faba bean (Khan et al., 2019). Moreover, the over-expression of the barley LEA HVA1 gene enhanced transgenic wheat biomass productivity and water use efficiency under water-limited conditions (Sivamani et al., 2000). Taken collectively, our results reveal that stress defense proteins, interacting with other proteins in complex networks, are essential for drought-tolerant ND476 maize plants’ survival under water-deficit conditions.

### The Gene Co-expression Network Analysis Offered an Essential Resource for Mining Novel Genes Related to Water-Deficit Stress Conditions

Given that the expression of a great number of DEGs was affected by drought treatment, WGCNA was used to construct a gene co-expression network to mine the major genes and dig out the key modules involved in the maize responses to drought stress from the vegetative to reproductive stages. In this study, a total of 12 modules were identified based on gene expression patterns, and several modules showed functional specificity in various stages, as genes were regulated dynamically under water-limited conditions (Figure 4). Then for investigating these DEGs’ functional biological roles, BiNGO software was used (Supplementary Table 5). The functions of DEGs with known biological functions could be predicted according to their module, and this analysis found a series of biological processes that were affected by drought stress. By comparing our results with previous studies, similar biological processes were identified in plants’ response to drought stress. Therefore, the gene co-expression network analysis provides an essential resource for mining novel genes related to water-deficit stress acclimation of maize. Particularly, the hub-genes are suggested to be the key players in maize drought stress response. Further downstream analysis studies will be essential in determining each of these hub genes‘ exact contribution to drought stress tolerance in maize.

### Proposed Molecular Model for Maize Drought Stress Tolerance

**FIGURE 8:**
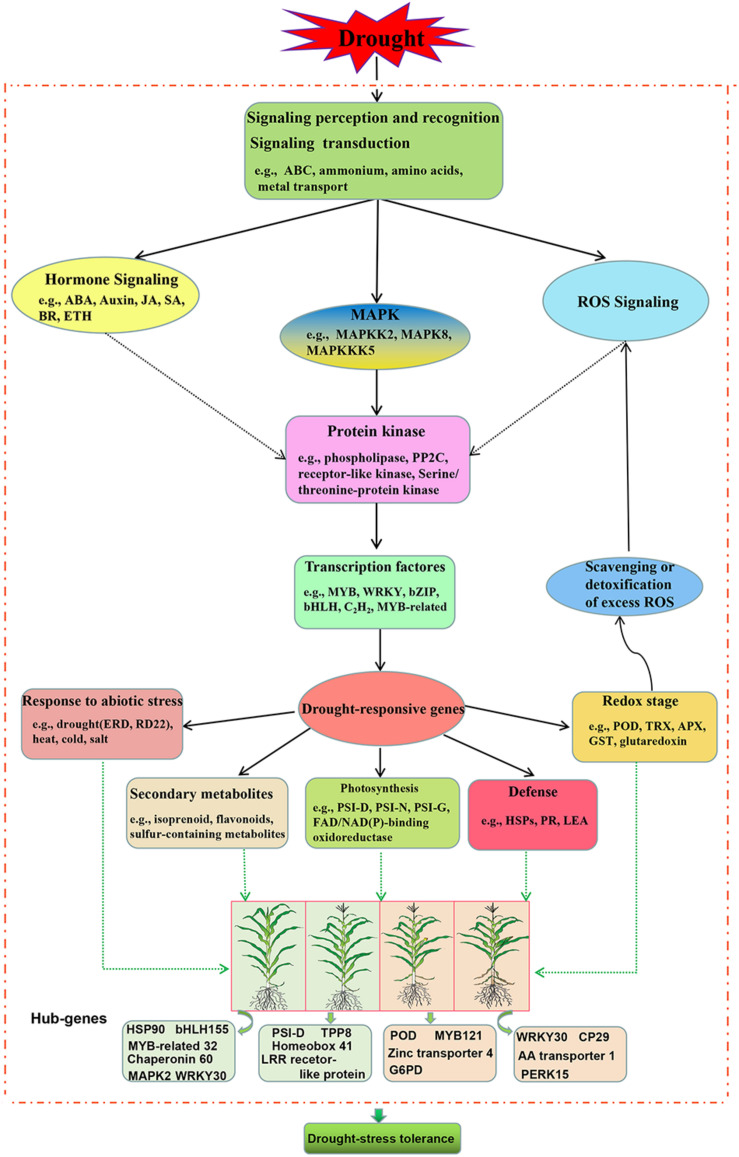
Model of the drought stress pathways occurring in maize from vegetative growth to reproductive development under drought stress. ABA, abscisic acid; JA, jasmonic acid; SA, salicylic acid; BR, brassinosteroid; ETH, ethylene; MAPK, mitogen-activated protein kinase; PP2C, protein phosphatase 2C; ERD4, early-response to dehydration stress; RD22, responsive to dehydration; POD, guaiacol peroxidase; TRX, thioredoxin; APX, ascorbate peroxidase; GST, glutathione S-transferases; HSP, heat shock proteins; PR, pathogenesis-related proteins; LEA, late embryogenesis proteins; PSI-D, photosystem I 20 kDa subunit II; PSI-N, photosystem I reaction center subunit N; PSI-G, photosystem I reaction center subunit V; PERK15, proline-rich receptor-like protein kinase 15; CP29, chlorophyll a-b binding protein CP29.1 chloroplastic.

According to our main findings of the drought-responsive DEGs and their related networks, in combination with the related relevant literature, we herein propose a molecular model for drought stress tolerance in maize at four different growth stages as shown in Figure 8.

## Conclusion

In the present study, we performed a comprehensive comparative leaf transcriptome analysis of the drought-tolerant maize hybrid ND476 plants subjected to water-sufficient (control) and water-deficit treatment conditions at four different growth stages. Based on the transcriptome analysis, a total of 3,451 DEGs were identified from the four experimental comparisons, and changes in these genes affected corresponding metabolic pathway responses related to drought tolerance in maize. Subsequently, 3,451 DEGs were divided into 12 modules by the WGCNA analysis. Our results showed that maize drought stress adaptation is a stage-specific response process. Whereas DEGs related to stress signal transduction, detoxification, transcription factor regulation, hormone signaling and secondary metabolites biosynthesis were universal across the four crop growth stages, those associated with photosynthesis and amino acid metabolism, protein degradation, transport, and RNA transcriptional regulation were uniquely enriched at the V12, VT, R2, and R4 stages, respectively. Our findings may help in clarifying the important growth-stage-specific molecular mechanisms regulating maize drought stress responses. Further, the key genes and metabolic pathways identified here may serve as valuable genetic resources or selection targets for genetic engineering of drought-resistant maize cultivars.

## Data Availability Statement

The datasets presented in this study can be found in online repositories. The names of the repository/repositories and accession number(s) can be found in the article/Supplementary Material.

## Author Contributions

SL, TZ, NW, and HD conceived and designed the experiments. SL, TZ, HD, AD, and YY performed the experiments. SL, TZ, AD, and YY analyzed the data. SL and TZ wrote the manuscript. All authors have read and approved the final manuscript.

## Conflict of Interest

The authors declare that the research was conducted in the absence of any commercial or financial relationships that could be construed as a potential conflict of interest.

## Footnotes

1 https://github.com/jstjohn/SeqPrep

2 https://github.com/najoshi/sickle

3 http://tophat.cbcb.umd.edu/

4 https://www.ncbi.nlm.nih.gov/

5 https://web.expasy.org/docs/swiss-prot

6 https://www.ncbi.nlm.nih.gov/COG/

7 http://www.genome.jp/kegg

8 http://www.geneontology.org

9 http://www.bioconductor.org/packages/2.12/bioc/html/edgeR.html

10 www.majorbio.com
