# Supplementary material for: Global Transcriptome and Weighted Gene Co-expression Network Analyses of Growth-Stage-Specific Drought Stress Responses in Maize
Source: Front Genet. 2021 Jan 26;12:645443. doi: 10.3389/fgene.2021.645443 (PMC7870802; doi:10.3389/fgene.2021.645443)
Supplement: Supplementary file 1 [file Data_Sheet_1.ZIP › Supplementary Material/Supplemantery Tables/Supplementary Table 1.doc]

**Supplementary Table 1.** Summary of Illumina RNA sequencing results for twenty-four samples isolated from maize leaves

| **Samples**^1^ | **Raw reads**^2^ | **Clean reads**^3^ | **Total mapped**^4^ | **Uniquely mapped**^5^ | **Q30(%)**^6^ | **GC content(%)**^7^ |
| --- | --- | --- | --- | --- | --- | --- |
| NDC1 | 48035096 | 47610098 | 43143631(90.62%) | 41372616(86.90%) | 96.33 | 55.80 |
| NDC2 | 54486068 | 54166538 | 50258591(92.79%) | 48336953(89.24%) | 96.56 | 57.05 |
| NDC3 | 44542680 | 44085504 | 38332401(86.95%) | 36372979(82.51%) | 96.50 | 54.60 |
| NDC4 | 42548972 | 41544188 | 35311007(85.00%) | 30342991(73.04%) | 94.50 | 55.95 |
| NDC5 | 40734384 | 39783580 | 35423813(89.04%) | 33861471(85.11%) | 94.46 | 55.80 |
| NDC6 | 41127584 | 40153870 | 35629542(88.73%) | 34012429(84.71%) | 94.51 | 55.30 |
| NDCA | 59925726 | 59580188 | 55088277(92.46%) | 51854517(87.03%) | 96.69 | 58.59 |
| NDCB | 73048054 | 72636578 | 67511922(92.94%) | 64399057(88.66%) | 96.71 | 57.78 |
| NDCC | 51946220 | 51558264 | 47579146(92.28%) | 45066907(87.41%) | 96.30 | 58.68 |
| NDCa | 55342254 | 54963524 | 50467490(91.82%) | 48503647(88.25%) | 96.44 | 57.27 |
| NDCb | 59161562 | 58848272 | 54571986(92.73%) | 52363482(88.98%) | 96.75 | 56.40 |
| NDCc | 58430550 | 58068856 | 53792336(92.64%) | 51774291(89.16%) | 96.62 | 56.59 |
| NDD1 | 57502078 | 57188372 | 53180180(92.99%) | 51087820(89.33%) | 96.62 | 56.84 |
| NDD2 | 60217732 | 59870224 | 55618716(92.90%) | 53420048(89.23%) | 96.48 | 56.63 |
| NDD3 | 54959348 | 54580992 | 49427404(90.56%) | 47440596(86.92%) | 96.56 | 56.35 |
| NDD4 | 27793124 | 27549536 | 25314496(91.89%) | 24277425(88.12%) | 95.54 | 55.15 |
| NDD5 | 47563710 | 46494702 | 41046147(88.28%) | 38920912(83.71%) | 94.64 | 55.33 |
| NDD6 | 23219294 | 23029648 | 21253143(92.29%) | 20432633(88.72%) | 96.15 | 55.61 |
| NDDA | 56338684 | 55977752 | 52008434(92.91%) | 49705362(88.79%) | 96.14 | 58.20 |
| NDDB | 59760736 | 59414606 | 55036131(92.63%) | 52216093(87.88%) | 96.63 | 57.84 |
| NDDC | 65312034 | 64915948 | 60089140(92.56%) | 56803851(87.5%) | 96.68 | 58.48 |
| NDDa | 54746454 | 54424602 | 50537680(92.86%) | 48566518(89.24%) | 96.63 | 57.66 |
| NDDb | 59668746 | 59322012 | 53685546(90.50%) | 51419976(86.68%) | 96.26 | 56.81 |
| NDDc | 56185708 | 55801650 | 51513215(92.31%) | 49344332(88.43%) | 96.45 | 56.89 |

^1^Samples, sample name of drought-tolerant genotype leaf tissues collected from four stages both of control and drought treatment; ^2^Raw reads, the reads amount before filtering; ^3^ Clean reads, the reads amount after filtering; ^4^ Total mapped:the percentage of mapped clean reads; ^5^Uniquely reads: the percentage of reads that map to only one location of the reference; ^6^Q30, the Q30 value for the raw bases/Calculate the percentage of bases that have greater than 30 Phred values; ^7^GC content: the ratio of GC bases for the raw bases.
