# Supplementary material for: Global Transcriptome and Weighted Gene Co-expression Network Analyses of Growth-Stage-Specific Drought Stress Responses in Maize
Source: Front Genet. 2021 Jan 26;12:645443. doi: 10.3389/fgene.2021.645443 (PMC7870802; doi:10.3389/fgene.2021.645443)
Supplement: Supplementary file 1 [file Data_Sheet_1.ZIP › Supplementary Material/Supplemantery Tables/Supplementary Table 2.doc]

**Supplementary Table 2.** Summary of the functional annotation of assembled genes

| **Database categories**^1^ | **Gene Number**^2^ | **Percentage**^3^ |
| --- | --- | --- |
| GO | 133525 | 69.41% |
| KEGG | 82758 | 43.02% |
| COG | 169691 | 88.21% |
| NR | 179093 | 93.10% |
| Swiss-Prot | 137849 | 71.66% |
| All | 192366 | 100% |

^1^Annotated database, the database used for functional annotation; ^2^Gene number, the number of genes predicted by the corresponding database`s annotation information; ^3^ Percentage, the percentage of annotated gene number to the total genes. GO, Gene Ontology; KEGG, Kyoto Encyclopedia of Genes and Genomes; COG, clusters of orthologous groups ; NR, non-redundant protein sequence; Swiss-Prot, a manually annotated protein sequence database; All, the total of differentially expressed genes in the data set/comparison group.
