# Supplementary material for: Global Transcriptome and Weighted Gene Co-expression Network Analyses of Growth-Stage-Specific Drought Stress Responses in Maize
Source: Front Genet. 2021 Jan 26;12:645443. doi: 10.3389/fgene.2021.645443 (PMC7870802; doi:10.3389/fgene.2021.645443)
Supplement: Supplementary file 1 [file Data_Sheet_1.ZIP › Supplementary Material/Supplemantery Tables/Supplementary Table 7.doc]

**Supplementary Table 7**. Gene specific primers used for qRT-PCR analysis

| Gene ID | Primer F（5’-3'） | Primer R（5’-3'） | TM（F) | TM（R) | Product |
| --- | --- | --- | --- | --- | --- |
| Zm00001d021647 | AGCCACCTATGCTGCTGTTT | CGCAAACGAATAGGACAGCG | 59.96 | 59.97 | 136 |
| Zm00001d042886 | TTGTCTGAGCTTGACGTGCT | ACCGGTGGCACAAACTTGA | 59.90 | 60.08 | 171 |
| Zm00001d025015 | CTGTAAGTTTGCACGCCCTC | TGCAAACTAGCCCTAACAGC | 59.48 | 58.18 | 151 |
| Zm00001d025831 | ATAGGAGGTGGGTGGTTGGG | GTGGGTGGGCAGATTCACAA | 60.92 | 60.54 | 120 |
| Zm00001d040399 | TTTTCTGCACCGCCTGGTAT | GGCAAGACAATCGAACACAGG | 59.96 | 59.80 | 184 |
| Zm00001d018220 | TCTGGGCTGACTTCATCGAC | ATGAATGTGCGTGAGGACAC | 59.47 | 58.56 | 92 |
| Zm00001d011132 | TGCTCACATTCATCGCCATCT | ACCCAATTCCAACCCACAGAG | 60.13 | 60.20 | 144 |
| Zm00001d043992 | AGTGGGGATCACAGCCTAGAA | TGTTGGCTAACTCGGAACATCA | 60.27 | 59.96 | 96 |
| Zm00001d051556 | GACGACAAGCCGACACAGAA | GATGTTGGATGGCCCTGACA | 60.60 | 60.03 | 192 |
| Zm00001d041853 | GCCACACCAACATGCTGTC | GCAGTGCAGAGGAGGTACAG | 59.71 | 60.11 | 150 |
| Zm00001d044812 | TCGCTTTTGGACTTTGCACG | AACGTCAGTCAGTGGCAGAG | 59.97 | 59.97 | 100 |
| Zm00001d024839 | TCCTCTCTATTGCTAGCTGCTT | GGCGTAGCCATATGTCCTGT | 58.70 | 59.61 | 183 |
