# Supplementary material for: Global Transcriptome and Weighted Gene Co-expression Network Analyses of Growth-Stage-Specific Drought Stress Responses in Maize
Source: Front Genet. 2021 Jan 26;12:645443. doi: 10.3389/fgene.2021.645443 (PMC7870802; doi:10.3389/fgene.2021.645443)
Supplement: Supplementary file 1 [file Data_Sheet_1.ZIP › Supplementary Material/Supplementary Figures.doc]

**Supplementary Figures (Total 7)**

A

B

**Figure S1**. Selection of weight Parameter β for constructing adjacency Matrix Network. (A) Scale independence tolerance curve, X axis denotes the exponentially weighted β value, Y axis denotes the fitting degree of the adjacent matrix and the scale-free network hypothesis for the corresponding β value. The larger R^2^ is, the more network conforms to the scale-free distribution. (B) Mean connectivity curve, X axis indicates the exponentially weighted β value, Y axis indicates the average connectivity (k/degree/connectivity) of each node in the network represented by the adjacency matrix corresponding to the transformed β value.

**Figure S2**. Leaf photosynthesis rate measurement at various crop growth (V12, VT, R2, and R4) stages (A-D). Data are means with n=3 plants. All the comparisons between water treatments and well-watered were significantly at * p <0.05, **p <0.01.

**Figure S3**. Intersample analysis of twenty-four leaf tissue samples used for transcriptome sequencing. (A) PCA analysis, after the sample is analyzed by dimension reduction, there are relative coordinate points on the principal component. The distance of each sample point represents the distance of the sample. The closer the distance, the higher the similarity between the samples. X axis represents the degree of contribution of a principal component N (PCN) in the two-dimensional graph to distinguish samples, Y axis represents the contribution of a principal component M (PCM) in the two-dimensional graph to distinguish samples.

**Figure S4**. Clustering analysis of DEGs shared by both vegetative and reproductive development stages. (A) the DEGs shared by R2 and V12 stages, (B) the DEGs shared by V12 and R4 stages, (C) the DEGs shared by VT and R4 stages, (D) the DEGs shared by R2 and R4 stages.

A

B

D

C

**Figure S5**. MapMan overview analysis of DEGs under various maize growth stages presenting the respective functional classifications. (A) V12 stage; (B) VT stage; (C) R2 stage; (D) R4 stage. The legend and the pie charts provide the parent BIN number, BIN name and the gene percentage in the parentheses.

**Figure S6**. Analysis of weighted gene co-expression network analysis (WGCNA) modules in which genes were enriched. Each module contains a statistical chart of the number of members. Horizontal coordinates represent the module (expressed in module color); vertical coordinates represent the number of members belonging to the module.

**Figure S7**. DEGs expression tendencies in both RNA-Seq data and qRT-PCR experimental results. The x-axis shows the result of qRT-PCR, and the y-axis represents the DEGs relative expression level.
